# A Semi-Automated Workflow for FAIR Maturity Indicators in the Life Sciences

**DOI:** 10.3390/nano10102068

**Published:** 2020-10-20

**Authors:** Ammar Ammar, Serena Bonaretti, Laurent Winckers, Joris Quik, Martine Bakker, Dieter Maier, Iseult Lynch, Jeaphianne van Rijn, Egon Willighagen

**Affiliations:** 1 Department of Bioinformatics—BiGCaT, NUTRIM, Maastricht University, NL-6200 MD Maastricht, The Netherlands; a.ammar@maastrichtuniversity.nl (A.A.); serena.bonaretti.research@gmail.com (S.B.); laurent.winckers@maastrichtuniversity.nl (L.W.); j.vanrijn@maastrichtuniversity.nl (J.v.R.); 2 Transparent MSK Research, NL-6221 BN Maastricht, The Netherlands; 3 National Institute for Public Health and the Environment (RIVM), NL-3720 BA Bilthoven, The Netherlands; joris.quik@rivm.nl (J.Q.); martine.bakker@rivm.nl (M.B.); 4 Biomax Informatics AG, 82152 Planegg, Germany; dieter.maier@biomax.com; 5 School of Geography, Earth and Environmental Sciences, University of Birmingham, Edgbaston, Birmingham B15 2TT, UK; i.lynch@bham.ac.uk

**Keywords:** FAIR guidelines, FAIR maturity indicators, life sciences, Jupyter Notebook

## Abstract

Data sharing and reuse are crucial to enhance scientific progress and maximize return of investments in science. Although attitudes are increasingly favorable, data reuse remains difficult due to lack of infrastructures, standards, and policies. The FAIR (findable, accessible, interoperable, reusable) principles aim to provide recommendations to increase data reuse. Because of the broad interpretation of the FAIR principles, maturity indicators are necessary to determine the FAIRness of a dataset. In this work, we propose a reproducible computational workflow to assess data FAIRness in the life sciences. Our implementation follows principles and guidelines recommended by the maturity indicator authoring group and integrates concepts from the literature. In addition, we propose a FAIR balloon plot to summarize and compare dataset FAIRness. We evaluated the feasibility of our method on three real use cases where researchers looked for six datasets to answer their scientific questions. We retrieved information from repositories (ArrayExpress, Gene Expression Omnibus, eNanoMapper, caNanoLab, NanoCommons and ChEMBL), a registry of repositories, and a searchable resource (Google Dataset Search) via application program interfaces (API) wherever possible. With our analysis, we found that the six datasets met the majority of the criteria defined by the maturity indicators, and we showed areas where improvements can easily be reached. We suggest that use of standard schema for metadata and the presence of specific attributes in registries of repositories could increase FAIRness of datasets.

## 1. Introduction

Data sharing and data reuse are two complementary aspects of modern research. Researchers share their data for a sense of community, to demonstrate integrity of acquired data, and to enhance the quality and reproducibility of research [1]. In addition, data sharing is supported by the emerging citation system for datasets, scientific journal requirements, and funding agencies that want to maximize their return on investments in science [2,3]. At the same time, researchers are eager to reuse available data to integrate information that answer interdisciplinary research questions and to optimize use of funding [4]. Although attitudes towards data sharing and reuse are increasingly favorable [1], data discovery and reuse remain difficult in practice [5]. Studies show that 40% of qualitative datasets were never downloaded, and about 25% of data is used less than 10 times [6]. In addition, Vines et al. demonstrated that the availability of existing datasets associated with published articles decreases 17% per year due to the lack of appropriate hardware to access old storage media or because data were lost [7]. To be effective, data sharing and reuse need appropriate infrastructure, standards, and policies [5].

In 2016, the FORCE 11 group proposed guidelines to increase data reuse in the life sciences. These guidelines aimed to make data findable, accessible, interoperable, and reusable, and were summarized with the acronym FAIR [8,9]. In a short time, the FAIR guidelines have gained remarkable popularity, and they are currently supported by funding agencies and political entities such as the European Commission, the National Institutes of Health in the United States, and institutions in Africa and Australia [10]. In addition, academic and institutional initiatives were launched to promote and implement data FAIRness, such as GO FAIR [11] and FAIRsharing [12]. 

Although largely adopted, the FAIR principles do not specify any technical requirement, as they are deliberately intended to be aspirational [10]. The lack of practical specifications generated a large spectrum of interpretations and concerns and raised the need to define measurements of data FAIRness [9]. Some of the authors of the seminal paper proposed a set of FAIR metrics [13], subsequently reformulated as FAIR maturity indicators [14]. At the same time, they invited consortia and communities to suggest and create alternative evaluators. The majority of the proposed tools are online questionnaires that researchers and repository curators can manually fill to assess the FAIRness of their data (Table 1). However, the FAIR metrics guidelines emphasize the importance of creating “objective, quantitative, [and] machine-interpretable” evaluators [13]. Following these criteria, two platforms have recently been developed to automatically compute FAIR maturity indicators: FAIR Evaluation Services and FAIRshake. The first platform offers an evaluation of maturity indicators and compliance tests [14], whereas the second platform provides metrics, rubrics, and evaluators for registered digital resources [15]. Both platforms provide use cases for FAIRness assessment, however they do not provide systematic analysis of evaluated datasets and repositories. Indeed, a key feature of the FAIR principles is their requirement for domain-specific identification of the metadata required to drive community acceptance and facilitate date reuse within and beyond that specific research community. An example of such a community-driven approach is presented by Papadiamantis et al. [16] on metadata standards for the nanosafety community, which extends the technical FAIR principles, which are directed largely at database managers and curators, with a further set of scientific FAIR principles directed at data generators (experimental and computational) defined to support operationalization of the FAIR principles for nanosafety researchers.

Literature reports two studies evaluating FAIRness for large datasets. Dunning et al. [17] used a qualitative approach to investigate 37 repositories and databases. They assessed FAIRness using a traffic-light rating system that ranges from no to full compliance. Weber et al. [18] implemented a computational workflow to analyze the retrieval of more than a million images from five repositories. They proposed metrics specific to images, including time and place of acquisition, to assess image provenance. The first study provides valuable concrete guidelines to assess data FAIRness, however the implementation was manual, diverging from what the guidelines suggest. The second study is a relevant example of computational implementation, although limited to retrieval of images and evaluation of 10 out of 15 FAIR principles, and without unique correspondence between the FAIR principles and the maturity indicators. 

These existing analyses show that more common features are easier to test, causing them to focus on the more common FAIR principles, particularly the findability and accessibility aspects, as these apply to any database or dataset. Then, analysis can be done at the repository/database level, which does not provide information about the FAIRness of the data that constitutes it, while the latter would decide if data can really be reused. Data quality aspects are part of the interoperability and reusability criteria and as such are underappreciated aspects of FAIR.

In this paper, we propose a computational approach to calculate FAIR maturity indicators in the life sciences as exemplified in nanotoxicology data. We followed the recommendations provided by the Maturity Indicator Authoring Group (MIAG) [14], and we created a visualization tool to summarize and compare FAIR maturity indicators across various datasets and/or repositories containing toxicology and/or nanotoxicology related data. We tested the feasibility of our approach on three real use cases where researchers retrieved data from six scientific repositories to answer their research questions. Finally, we made our work open and reproducible by implementing our computations in a Jupyter Notebook using Python.

## 2. Materials and Methods 

### 2.1. Use Cases in the Life Sciences

We asked researchers in the Department of Bioinformatics (BiGCaT) at Maastricht University for cases where they looked for datasets in a scientific repository to answer a specific research question. Each use case is labeled with a name that is used throughout the paper. The use cases names, research questions, and investigated repositories are:

- *Parkinsons_AE*: What are the differentially expressed genes between normal subjects and subjects with Parkinson’s diseases in the brain frontal lobe? To answer this question, the researcher looked for a dataset in the search engine of ArrayExpress (AE), a repository for microarray gene expression data based at the European Bioinformatics Institute (EBI), United Kingdom [19];
- *NBIA_GEO*: What is the effect of the *WDR45* gene mutation in the brain? In this case, the researcher looked for a dataset in the search engine of Gene Expression Omnibus (GEO), a repository containing gene expression and other functional genomics data hosted at the National Center for Biotechnology Information (NCBI), United States [20].
- *TiO_2__eNanoMapper, TiO_2__caNanoLab, TiO_2__ChEMBL* and *TiO_2__NanoCommons*: What can these datasets tell us about nanoscale titanium dioxide (TiO_2_) toxicity? Toxicological studies have shown that TiO_2_ nanoparticles (NPs) induce oxidative stress in cells resulting in immune response, inflammation, genotoxicity, and cell damage [21]. The researcher looked for a dataset in the search engine of four databases eNanoMapper [22], caNanoLab [23], ChEMBL [24], and NanoCommons [25] for data related to TiO_2_ induced cytotoxicity, immunotoxicity, genotoxicity, or oxidative stress.

### 2.2. What is Data and What Is Metadata?

The FAIR principles (listed in Table 2) use the terminology *data*, *metadata*, and *(meta)data*. For our computational implementation, we needed precise definitions of these terms:

- *data*: According to the Merriam-Webster online dictionary, *data* are “information in digital form that can be transmitted or processed” [26];
- *metadata*: In the Merriam-Webster online dictionary, *metadata* are defined as “data that provide information about other data” [27];
- *(meta)data:* We interpreted it as *data and/or metadata*. We used *(meta)data* as:
  ∘ *data* for the principles R1, R1.1, and R1.2;
  ∘ *metadata* for the principles I1 and I3;
  ∘ *data and metadata* for the principles F1, F4, and A1.

In our implementation, these terms assumed the following meanings:

- data: It is the actual dataset that researchers analyzed to answer their research question. The analysis of the dataset itself is out of the scope of this study;
- *metadata: For the following principles, the corresponding metadata are:*
  ∘ F2: Information that allows researchers to find the dataset s/he looks for. It coincides with the keywords used in the search;
  ∘ F3: Identifier of the dataset in the repository;
  ∘ I3: Reference to other metadata;
  ∘ R1: Information about the dataset, other than the search keywords;
  ∘ R1.1: Data license;
  ∘ R1.2: Data provenance as publication title, author names, and one author’s email address.

In all cases, we assumed that *data* and *metadata* were hosted in the same repository.

### 2.3. Calculating FAIR Maturity Indicators

Because the FAIR guidelines emphasize the importance of *data* and *metadata* being “machine-interpretable”, we collected information about datasets and repositories via application programming interfaces (API) wherever possible. We queried six different sources:

- Data repositories (ArrayExpress, Gene Expression Omnibus, eNanoMapper, caNanoLab, ChEMBL, and NanoCommons): We programmatically queried each repository using the same keywords researchers had used in their manual query when looking for a dataset. From the obtained metadata, we retrieved information to calculate maturity indicators for the principles F2, F3, I1, I3, R1, and R1.2 (see Table 2); The aforementioned principles are assessed directly on the data repository without relying on external sources like registries as in the next point.
- Registry of repositories: We queried re3data.org, a registry containing information about more than 2000 data repositories from various disciplines. We used the retrieved information to compute the maturity indicators for the principles F1, A2, and R1.2; F1 was chosen to be assessed from an external source, because it can be performed in an automated way. re3data.org provides information on whether the data repository provides a persistent unique identifier for its data or not. Obtaining such information from the data source itself is not possible. The same goes for metadata policy (A2) and metadata provenance (R1.2): we need a registry that can be queried programmatically to obtain such information. Otherwise, data repositories can provide this information within unstructured text, and it is not feasible to find it in an automated way.
- Searchable resource: We queried Google Dataset Search, an emerging search engine specific for datasets, to quantify the principle F4, which relates to indexing of the metadata in a searchable resource.

The output of queries consisted of information structured in XML or JSON. Details about the computation of each specific maturity indicator are provided in Table 2, in the *Availability* section below, and on our companion website. To each maturity indicator, we assigned binary value 1 if the criterion was satisfied and 0 in the opposite case, with the exception of indicators F2 and R1.2. The former was calculated as the ratio between the number of keywords in the metadata over the total number of keywords used by the researcher in the manual query, while the latter was given a score of 0.5, 0, or 1 if, respectively, one/two out of three provenance details, none, or all details were provided.

Similar to previous studies [17,18,28], we did not evaluate maturity indicators for the principles I2 and R1.3.

### 2.4. Visualizing FAIR Maturity Indicators

To summarize and compare FAIRness of datasets, we developed the FAIR balloon plot using the R package ggplot2 [29]. In the graph, each row corresponds to a use case and each column to a FAIR maturity indicator. The size of each shape is the value of a specific FAIR maturity indicator for a particular dataset. Diamonds represent maturity indicators determined manually, circles depict maturity indicators established automatically, and crosses illustrate the maturity indicators we did not compute. Finally, colors represent the group of principles in the FAIR acronym: blue for findable, red for accessible, green for interoperable, and orange for reusable.

## 3. Results

**Figure 1:**
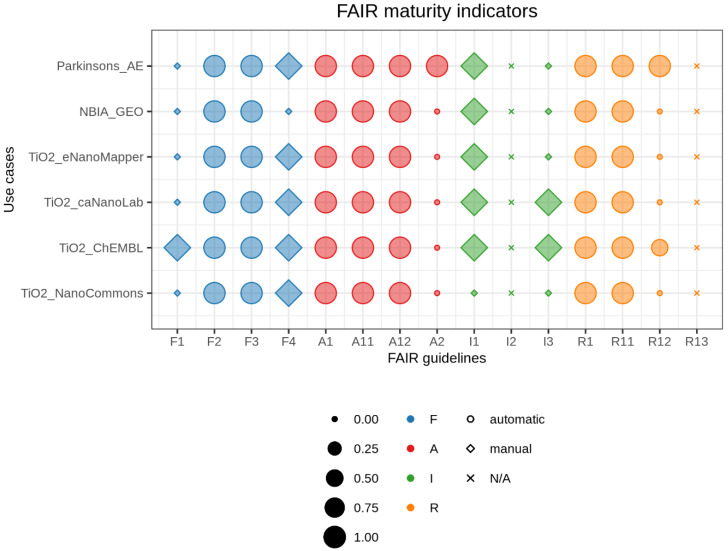
FAIR balloon plot. Comparative summary of FAIR maturity indicators for the six databases evaluated in this work. Size corresponds to the numerical value of mutual indicators, colors represent FAIR categories, and shapes illustrate the way we retrieved information (N/A = not available). The graph can be fully reproduced from our Jupyter Notebook on GitHub.

A comparative summary of the results is shown in Figure 1 and details of the findings can be found in Supplementary Information Table S1. For all use cases, the metadata contained (i) all keywords used in the manual search (F2), (ii) dataset unique identifiers (F3), and (iii) additional information for data reuse (R1). In addition, they were structured in either XML or JSON format (I1) and were released with a clear usage license (R1.1). The protocol used to retrieve all information was HTTP, which is standardized (A1), open, free, and universally implementable (A1.1), and allows for authentication where needed (A1.2). Apart from ArrayExpress, none of the databases provided a policy to keep the metadata accessible if the data itself is no longer available (A2). In all cases except *TiO_2__ChEMBL*, metadata was not assigned a persistent identifier (F1), and only *TiO_2__caNanoLab* and *TiO_2__ChEMBL* referenced other metadata (I3). Finally, all databases except NBIA_GEO were listed in Google Dataset Search (F4), while only the datasets of the *Parkinsons_AE* and *TiO_2__ChEML* use cases had detailed provenance (R1.2). Note that *TiO_2__ChEMBL* scored 0.5 points for R1.2, since only part of the provenance details were provided. As stated in the Methods section, maturity indicators for the principles I2 and R1.3 were not evaluated.

## 4. Discussion

We proposed a semiautomatic computational approach to evaluate FAIR maturity indicators for scientific data repositories in the life sciences. We tested the feasibility of our method on three real use cases where researchers looked for datasets to answer their scientific questions. Despite having different data types and difference purposes, the three use cases, for six databases, scored similarly. Finally, we created a FAIR balloon plot to summarize and compare our results, and we made our approach open and reproducible. Real use cases in the life sciences were the starting point of our computational implementation.

In their guidelines, the MIAG suggests to calculate maturity indicators starting from a global unique identifier (GUID) (e.g., InChI, DOI, Handle, URL) [28]. However, *a priori* knowledge of a GUID often signifies that a researcher has already found and accessed the dataset they are going to reuse. In addition, it assumes that the repository of interest provides unique identifiers, which is not the case for all the databases assessed in this work, based on the information we retrieved from re3data.org. Similar to Weber et al. [18], we decided to start our computations from dataset retrieval. We explored how researchers looked for the datasets of interest and which keywords they used, e.g., as part of the eNanoMapper requirement analysis [30]. Then, we computationally reproduced their manual search by programmatically retrieving data and metadata using the same keywords. We recognize that this approach limits the generalization of the FAIRness calculation. We note that the definition of FAIR, in fact, is different from one use case to another. While creating a use case for every dataset is extremely demanding, the same dataset could be used to answer different research questions.

To assess data FAIRness, we implemented criteria that follow principles and guidelines recommended by the MIAG [28], reused concepts from similar studies in literature [17,18], and added new considerations (see Table 2):

- *Findability*: The criteria to assess principles F1 (unique identifier), F3 (metadata includes identifier), and F4 ((meta)data are indexed) are similar for all previous studies. In our case, to assess F1 we investigated whether a repository provides a DOI in the registry re3data.org. We chose this registry because it is one of the largest registries of scientific repositories, and it provides an open API. Of course, different communities use different approaches, and FAIRSharing is an important complementary service [12]. For F3, we accepted any dataset identifier provided by the repository as the principle does not explicitly mention restrictions on the characteristics of the identifier. Finally, for F4 we looked for dataset titles in Google Dataset Search. We chose this searchable resource because it could become one of the main search engines specific for data in the future, similar to Google Scholar for publications. However, for Google Dataset Search or the newer DataCite Commons (https://commons.datacite.org/) to recognize datasets, the datasets also need semantic annotation, with, for example, schema.org. This is not tested in the current notebook. Another limitation is that, in contrast to the previous maturity indicators, the implementation of F2 (data are described with rich metadata) has large variations across literature publications. The MIAG recommends to evaluate whether metadata contains “structured” elements, Dunning et al. looked for attributes that favor findability, whereas Weber et al. used metrics of time and space of image acquisition. We followed the criteria suggested by Dunning et al. and looked for the keywords that researchers had used in their manual search to *find* datasets.
- *Accessibility*: Similar to the other published approaches, we retrieved our data using the HTTP protocol, which is free, open, and allows for authentication, and thus satisfies all the requirements of the A1 group. Additionally, there is concordance among approaches for the principle A2, which requires that a repository should explicitly provide a policy for data availability. In our implementation, we looked for the policy in re3data.org. However, for integration into research workflows, the mere use of the HTTP is a very narrow definition and choices of protocols on top of HTTP may be needed, e.g., for the authentication.
- *Interoperable*: Similarly to the MIAG, we assigned a positive score to metadata in a structured file format, such as XML (I1). In contrast, Dunning et al. and Weber et al. suggested that metadata should be in a standardized schema, such as Dublin Core or DataCite, which would increase data interoperability and simplify retrieval. None of the studies assessed I2 (vocabularies are FAIR), because it would require a separate implementation that includes the recursive nature of the FAIR principles. Finally, for I3 all previous studies looked for references to other datasets in metadata. Similarly to accessibility, these metrics are only a first step and not enough to link the various information sources needed to apply workflows for risk governance.
- *Reusable*: Although the MIAG does not provide any guidelines, the various studies implemented different ways to assess R1 (plurality of relevant attributes). While Weber et al. used the same metrics as for F2, Dunning et al. focused on metadata that provide information on how to reuse a dataset. In our implementation, we assess the presence of metadata attributes other than search keywords. The principles R1.1 (availability of data usage license) and R1.2 (data provenance) had a straight-forward implementation for all approaches. In our approach, we looked for a data license in re3data.org and for authors, author emails, and titles of the corresponding publication in the metadata from the dataset repository. Note that data would ideally be shared before publication and arguably should be shared as independent research output, in which case our implementation of R1.2 would not suffice. Finally, none of the authors evaluated whether metadata follow community standards (R1.3), as community agreements are not formally established yet. It will be clear that here too that these minimal expectations are not a sufficient requirement to ensure research output is practically useful for risk assessment.

We assessed FAIR maturity indicators using a mixed manual and automatic approach. In the literature, Dunning et al. used a fully manual approach to assess the maturity indicators, whereas Weber et al. used a completely automatic approach, calculating 10 of the total 15 maturity indicators. Our mixed approach enabled automatic assessment of maturity indicators wherever possible, and us to manually complement when we could not retrieve information via API. By definition, that means none of the databases could reach a full FAIRness score, since not all information was automatically retrieved. 

As repositories do not use a standardized metadata schema, our mixed implementation required prior manual investigation of metadata attributes for each repository. For example, ArrayExpress uses the attributes “authors”, “email”, and “title” that we could use for the principle R1.2, whereas ChEMBL uses only the “authors” and “title”, making the score of principle R1.2 0.5 points for not providing the full provenance details. Finally, Gene Expression Omnibus, eNanoMapper, caNanoLab, and NanoCommons do not have attributes for provenance. Clearly, community standards for data and metadata must be specified, in addition to minimal reporting standards and data quality criteria. This is a significant challenge for a cross-disciplinary field such as nanosafety. A first approach to build consensus in terms of the requirements for a nanosafety metadata schema [16] has been developed via the Nanomaterial Data Curation Initiative (NDCI), a project of the National Cancer Informatics Program Nanotechnology Working Group (NCIP NanoWG) [31]. Part of the challenge lies in the large variety of guidelines and data requirements that play a role in risk assessment of different nanomaterial applications, e.g., in the food, pharmaceutical, and other industrial sectors, and the very broad range of data reuse scenarios making complete metadata description an extensive task. 

Established minimal reporting standards and data quality measures for nanosafety data as developed by the European and US nanoinformatics communities, on the other hand, are currently not defined as FAIR metrics [32], nor are experiment-specific standards like MIAME for microarray experiments [33], CONSORT-AI for clinical trials [34], and MINBE for nanomaterial biocorona experiments [35]. It should also be noted that data quality and data completeness, unlike reporting standards, are dependent on the goal of use of the dataset [36]. For instance, two studies derived expectations related to data completeness as part of their assessment of nanosafety data quality [37,38]. Both include material properties, but Comandella and co-workers specifically included the completeness of metadata for the different methods applied to measure the different nanomaterial physico-chemical properties (chemical composition, size, surface charge, etc.), because this is important for read-across applications [37]. The study by Fernandez-Cruz et al. limited its assessment to whether these physico-chemical properties were reported, focusing on the data rather than the metadata as relevant for risk assessment [38]. These studies also show that it should be possible to derive metrics for data quality related to different study goals. 

It is important to note, however, that compliance with these established standards and quality measures, in addition to compliance to FAIR principles, still does not ensure that data can actually be (re)used in risk assessment, for example by the forthcoming Risk Governance Council for nanomaterials [39]. It should not be forgotten that regulatory agencies also apply criteria for assessing reliability, relevance, and consistency of data to be used in risk assessment [40,41]. These types of guidelines should all be considered relevant in establishing community standards for assessing data quality and extending the current set of FAIRness metrics, as part of the domain-relevant community standards, as defined in R1.3.

Before we can update an automated analysis of the relevant FAIRness scores, the relevant quality metrics first must be selected and formally defined, as discussed in the previous paragraphs. We note here that the selection of these metrics is likely to differ from one application to another. For example, nanoQSAR approaches may have different requirements than a read-across application. Note that even when data are of exceptional quality, this does not warrant their direct application in risk assessment if they are not transformed into parameters required in risk assessment models and tools, such as Benchmark Dose, EC50 or half-life in the environment, although mechanistic data can be used as part of a weight of evidence approach in this case.

**Figure 2:**
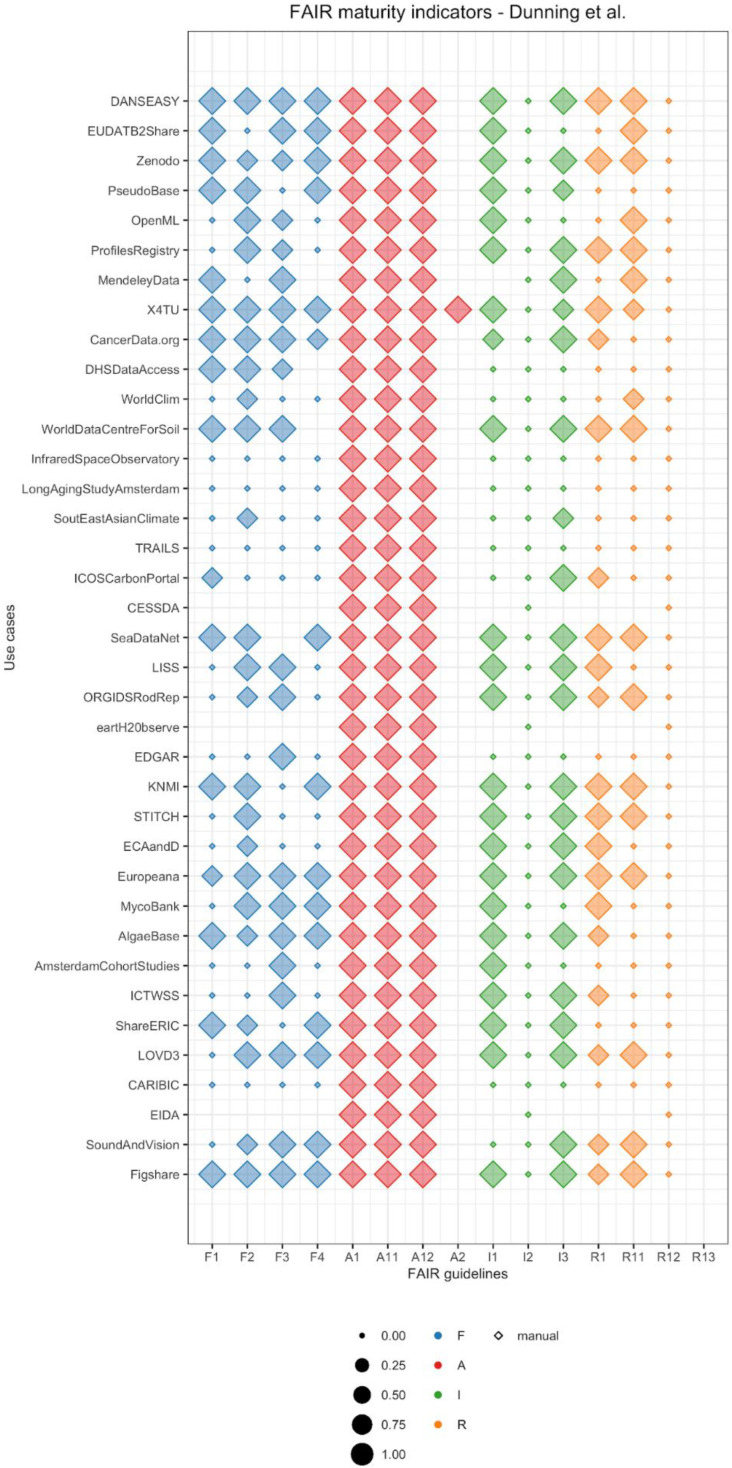
FAIR balloon plot for the repositories analyzed by Dunning et al. [17] (data available at their institutional repository). From their quantitative scores, we converted “complies completely” to 1, “just about/maybe not” to 0.5, and “fails to comply” to 0. We did not assign any value to “unclear”, which is thus represented as missing elements. The graph can be fully reproduced from our Jupyter Notebook on GitHub and interactively in binder.

Besides the limitations of the current FAIRness metrics noted above, they serve well as a screening tool to evaluate the level of FAIRness of existing life science and nanosafety databases. Only then can we start defining where to begin with making our resources more FAIR. For this, to summarize and compare dataset FAIRness, we created a FAIR balloon plot. As the MIAG guidelines recommend, we did not create a final score to avoid concerns for data and resource providers [14]. In our visualization, a dataset that reached full FAIRness (at the level measured by the used metrics) would have all maturity indicators depicted as circles (or diamonds in the case of manually determined metrics) with maximum size, meaning full score and automatic retrieval. In addition, by vertically stacking representations for different datasets, we can visually compare FAIRness levels for each maturity indicator. In the literature, another example of visualization is *insignia*, created for the platform FAIRshake [15]. It consists of multiple squares colored from blue (satisfactory) to red (unsatisfactory) for different levels of FAIRness. In addition, they can dynamically expand to visualize multiple scores calculated using different rubrics (i.e., criteria). Although this representation embeds the possibility of using different criteria, it does not allow direct comparison across datasets. Finally, we applied our FAIR balloon plot to the results collected by Dunning et al. to demonstrate that this kind of visualization can be reused for FAIR assessment with other criteria (Figure 2).

Furthermore, to make our analysis open and reproducible, we implemented our approach in a Jupyter Notebook. This shows the exact details of how the FAIRness is assessed, which we anticipated could help the developers of the databases to improve their FAIRness. However, changes to APIs or metadata attributes could affect reproducibility of the results. The possibility of querying a specific version of a repository could be a possible solution. In addition, we implemented our approach in Python, a language increasingly used in various scientific communities that can potentially favor extension and reuse of our work. For new datasets, FAIR maturity indicators could be evaluated by changing the search procedure and the values assigned manually. However, our observation is that the diversity in choices of protocols, standards, and other approaches to FAIR makes the possibility of a unifying approach remote. Another limitation of this approach is that it tests databases in a single way, whereas databases can have multiple, complementary access routes, each with their own use case and own level of FAIRness.

The six analyzed datasets met the majority of the criteria used to assess FAIRness, with ChEMBL being relatively the most FAIR dataset and GEO the least FAIR. Higher FAIRness compliance could be reached by using a standard for metadata (e.g., Dublin Core, DataCite, or schema.org), which could include all attributes required by the FAIR principles, and by providing explicit information about data policy, licenses, etc., to registries of repositories.

## 5. Conclusions

We propose a reproducible computational workflow to assess data FAIRness in the life sciences, and we created a FAIR balloon plot to summarize and compare FAIRness compliance. We evaluated our approach on three real cases, one of which was applied to 4 unique databases, i.e., the TiO_2_ nanomaterials’ toxicity assessment, and we demonstrated that the FAIR balloon plot can be extended to other FAIRness analyses. Finally, we suggest that use of standard schema for metadata and the presence of specific attributes in registries of data repositories could increase FAIRness of datasets with minimal additional effort. Nevertheless, to reach the interoperability needed to make nanosafety data really reusable, we first need to establish a much richer collection of FAIR metrics that also include metrics that reflect the domain-relevant community standards (R1.3). That should be the future focus point in FAIRification of nanosafety data.

## Figures and Tables

**Table 1:** Online FAIR (findable, accessible, interoperable, and reusable) evaluators and studies in the literature, which assess FAIRness of data repositories (the symbol ✓ indicates “yes”, the symbol - indicates “no”).

Authors	Questionnaire/Platform	Manual Assessment	Automatic Assessment			Data/Code Repository
-	-	-	Code/Language	Metadata Format	Protocol/Library	
**FAIRness evaluators**						
Wilkinsons et al. [13]	-	✓	-	-	-	GitHub
Australian Research Data Commons	FAIR self-assessment tool	✓	-	-	-	-
Commonwealth Scientific and Industrial Research Organization	5 star data rating tool	✓	-	-	-	-
Data Archiving and Networked Services	FAIR enough? and FAIR data assessment tool	✓	-	-	-	-
GOFAIR consortium	FAIR ImplementationMatrix	✓	-	-	-	Open Science Framework
EUDAT2020	How FAIR are your data?	✓	-	-	-	Zenodo
Wilkinsons et al. [14]	FAIR evaluation services	-	Ruby on Rails	JSON, Microformat, JSON-LD, RDFa	nanopublications	GitHub
Clark et al. [15]	FAIRshake	-	Django and Python	RDF	Extruct	GitHub
**Studies assessing FAIRness of repositories**						
Dunning et al. [17]	-	✓	-	-	-	Institutional repository
Weber et al. [18]	-	-	Python	DataCite	OAI-PMH	GitLab
Our approach	-	✓ (partially)	Jupyter notebook with Python	XML, JSON	request	GitHub

**Table 2:** FAIR principles and corresponding evaluation criteria proposed by the Maturity Indicator Authoring Group [28], Dunning et al. [17], Weber et al. [18], and our approach. The criteria used in the first two works are extracted from their publication text, whereas the criteria of Weber et al. are from Table IV of their paper. The metrics Weber et al. developed are Q_geo_ for image location, Q_time_ for the time of picture acquisition, Q_ret_ when data is automatically downloadable only given its metadata, and Q_lic_ for found license. In our approach, *dataset* metadata refers to metadata retrieved from the six databases used in our use cases, whereas *registry* metadata consists of metadata retrieved from re3data.org. In addition, we specify use of *(meta)data* as (data), (metadata), or (data and metadata), and automatic (A) or manual (M) procedure to retrieve information. Acronyms: GUID = Globally Unique IDentifier, DOI = Digital Object Identifier.

FAIR Principles [8]	Guidelines by the Maturity Indicator Authoring Group [28]	Dunning et al. [17]	Weber et al. [18]	Our Approach
F1: (meta)data are assigned a globally unique and persistent identifier	The GUID matches a scheme that is globally unique and persistent in FAIRsharing	Persistent identifier is DOI or similar	Pass (embedded in DataCite)	“doi” icon is enabled in www.re3data.org (data and metadata) (M)
F2: data are described with rich metadata (defined by R1 below)	Metadata contains “structured” elements (micrograph, JSON) or linked data (JSON-LD, RDFa)	Title, creator, date, contributors, keywords, temporal and spatial coverage	Q_geo_, Q_chrono_	Search keywords are in *dataset* metadata (A)
F3: metadata clearly and explicitly include the identifier of the data they describe	Metadata contains both its own GUID and the data GUID	DOI of data is in metadata	Pass (embedded in DataCite)	*Dataset* metadata contains dataset ID (A)
F4: (meta)data are registered or indexed in a searchable resource	The digital resource can be found using web-based search engines	Dataset title found in google.com or duckduckgo.com	Pass	Dataset title found in Google Dataset Search (data and metadata) (M)
A.1 (meta)data are retrievable by their identifier using a standardized communications protocol	N/A	HTTP request returns 200	Q_ret_	HTTP request returns 200 (data and metadata) (A)
A1.1 the protocol is open, free, and universally implementable	The resolution protocol is universally implementable with an open protocol	Accomplished if protocol is HTTP	Q _ret_	Accomplished if protocol is HTTP (A)
A1.2 the protocol allows for an authentication and authorization procedure, where necessary	The resolution protocol supports authentication and authorization for access to restricted content	Accomplished if protocol is HTTP	Q_ret_	Accomplished if protocol is HTTP (A)
A2. metadata are accessible, even when the data are no longer available	There is a policy for metadata	Repository has a clear policy statement	N/A	“data availability policy” is filled in *registry* metadata (A)
I1. (meta)data use a formal, accessible, shared, and broadly applicable language for knowledge representation	If hash-style metadata (e.g., JSON) or Linked Data are found, pass	Metadata is structured (e.g., Dublin Core)	Pass (embedded in DataCite)	*Dataset* metadata is structured (e.g., xml) (metadata) (M)
I2. (meta)data use vocabularies that follow FAIR principles	(meta)data use vocabularies that are, themselves, FAIR	N/A	N/A	N/A
I3. (meta)data include qualified references to other (meta)data	Metadata contain links that are not from the same source (domain/host)	Links to publications and terms definitions	N/A	*Dataset* metadata include reference to other dataset IDs (metadata) (M)
R1. meta(data) are richly described with a plurality of accurate and relevant attributes	N/A	Metadata provide information on how to reuse a dataset	Q_geo_, Q_chrono_	*Dataset* metadata contain more information than search keywords (F2) (data) (A)
R1.1. (meta)data are released with a clear and accessible data usage license	Metadata contain a pointer to the data license	Metadata license is present	Q_lic_	“datalicensename” and “datalicenseurl” are filled in *registry* metadata (data) (A)
R1.2. (meta)data are associated with detailed provenance	N/A	Documentation on how data was created	N/A	“authors”, “email” and “title” are filled in *dataset* metadata (data) (A)
R1.3. (meta)data meet domain-relevant community standards	N/A	N/A	N/A	N/A

## Supplementary Materials

The following are available online at https://www.mdpi.com/2079-4991/10/10/2068/s1, Table S1: Comparison of API systems and FAIR maturity indicators for the use cases analyzed in this work.

## Availability

The Jupyter Notebooks are provided in two GitHub repositories, one more general and one specific to the NanoSafety Cluster: https://github.com/BiGCAT-UM/FAIR_metrics and https://github.com/NanoCommons/wgf-fairdata-analysis. We further set up a website to summarize the current results, including the links to MyBinder to open the notebooks: http://nanocommons.github.io/wgf-fairdata-analysis/.
