# Supplementary material for: A Semi-Automated Workflow for FAIR Maturity Indicators in the Life Sciences
Source: Nanomaterials (Basel). 2020 Oct 20;10(10):2068. doi: 10.3390/nano10102068 (PMC7594074; doi:10.3390/nano10102068)
Supplement: Supplementary file 1 [file nanomaterials-10-02068-s001.zip › nanomaterials-937094-supplementary.docx]

A Semi-Automated Workflow for FAIR Maturity Indicators in the Life Sciences

Ammar Ammar ^1,†^, Serena Bonaretti ^1,2,†^, Laurent Winckers ^1^, Joris Quik ^3^, Martine Bakker ^3^, Dieter Maier ^4^, Iseult Lynch ^5^, Jeaphianne van Rijn ^1^, and Egon Willighagen ^1,^*

^1^ Department of Bioinformatics - BiGCaT, NUTRIM, Maastricht University, NL-6200 MD Maastricht, Netherlands; a.ammar@maastrichtuniversity.nl (A.A.); serena.bonaretti.research@gmail.com (S.B.); laurent.winckers@maastrichtuniversity.nl (L.W.); j.vanrijn@maastrichtuniversity.nl (J.V.R.)

^2^ Transparent MSK Research, NL-6221 BN Maastricht, Netherlands (<https://tmskr.github.io/>)

^3^ National Institute for Public Health and the Environment (RIVM), NL-3720 BA Bilthoven, Netherlands; joris.quik@rivm.nl (J.Q.); martine.bakker@rivm.nl (M.B.)

^4^ Biomax Informatics AG, 82152 Planegg, Germany; dieter.maier@biomax.com

^5^ School of Geography, Earth and Environmental Sciences, University of Birmingham, Edgbaston, Birmingham B15 2TT, UK; i.lynch@bham.ac.uk

***** Correspondence: [egon.willighagen@maastrichtuniversity.nl](mailto:egon.willighagen@maastrichtuniversity.nl)

**^†^** Both authors contributed equally to the manuscript

**Table S1.** Comparison of API systems and FAIR maturity indicators for the uses cases analyzed in this work. For each maturity indicator, we indicate the outcome in natural language and in numbers (1 or 0.5 for pass and 0 for fail).

| Use case | Parkinsons_AE | NBIA_GEO | TiO2_eNanoMapper | TiO2_caNanoLab | TiO2_ChEMBL | TiO2_NanoCommons |
| --- | --- | --- | --- | --- | --- | --- |
| Repository / Database | [Array Express](https://www.ebi.ac.uk/arrayexpress/) | [Gene Expression Omnibus](https://www.ncbi.nlm.nih.gov/geo/) | [eNanoMapper](https://www.ncbi.nlm.nih.gov/pubmed/26425413) | [caNanoLab](https://www.nature.com/articles/npre.2010.5175.1) | [ChEMBL](https://www.ncbi.nlm.nih.gov/pmc/articles/PMC3245175/) | NanoCommons |
| Search output on browser | [link](https://www.ebi.ac.uk/arrayexpress/experiments/E-MTAB-1194/) | [link](https://www.ncbi.nlm.nih.gov/geo/query/acc.cgi?acc=GSE70433) | [link](https://api.ideaconsult.net/enanomapper/select?rows=1000&wt=json&q=titanium%20dioxide) | [link](https://cananolab.nci.nih.gov/caNanoLab/#/sampleResults) | [link](https://www.ebi.ac.uk/chembl/api/data/document/search?format=json&q=titanium%20dioxide) | [link](https://ssl.biomax.de/info/biomax/bin/view/BioXM/WebServices) |
| API |  |  |  |  |  |  |
| Type | REST | REST | REST | REST | REST | REST |
| Documentation | [link](https://www.ebi.ac.uk/arrayexpress/help/programmatic_access.html) | [link](https://www.ncbi.nlm.nih.gov/geo/info/geo_paccess.html) | [link](http://ambit.sourceforge.net/enanomapper_usage.html) | [link](https://wiki.nci.nih.gov/display/caNanoLab/caNanoLab+User%27s+Guide) | [link](https://www.ebi.ac.uk/chembl/api/data/docs) | [link](https://ssl.biomax.de/info/biomax/bin/view/BioXM/WebServices) |
| Output format | XML | XML | JSON | JSON | JSON | JSON |
| FAIR maturity indicators |  |  |  |  |  |  |
| F1 (Persistent identifier) | No (0) | No (0) | No (0) | No (0) | Yes (1) | No (0) |
| F2 (Findable metadata) | parkinson’s disease, normal, homo sapiens, transcription profiling by array, raw data, frontal lobe, male, female (1) | nbia, homo sapiens, expression profiling by array (1) | TiO2 (1) | titanium dioxide (1) | titanium dioxide (1) | TiO2 (1) |
| F3 (Unique identifier) | 219251 (1) | 200070433 (1) | NRG2-0ee596a2-1bb4-3f56-b61e-25c660898570/5298 (1) | 86507520 (1) | CHEMBL1157425 (1) | NP00254 (1) |
| F4 (Google Dataset Search) | Yes (1) | No (0) | Yes (1) | Yes (1) | Yes (1) | Yes (1) |
| A1 (Communication protocol) | request status code = 200 (1) | request status code = 200 (1) | request status code = 200 (1) | request status code = 200 (1) | request status code = 200 (1) | request status code = 200 (1) |
| A1.1 (Open and free protocol) | Yes (1) | Yes (1) | Yes (1) | Yes (1) | Yes (1) | Yes (1) |
| A1.2 (Communication protocol) | Yes (1) | Yes (1) | Yes (1) | Yes (1) | Yes (1) | Yes (1) |
| A2 (Metadata always accessible) | Yes: <https://www.ebi.ac.uk/arrayexpress/help/data_availability.html> (1) | No (0) | No (0) | No (0) | No (0) | No (0) |
| I1 (Language representation) | XML (1) | XML (1) | JSON (1) | JSON (1) | JSON (1) | NA (0) |
| I2 (FAIR vocabularies) | Not evaluated (None) | Not evaluated (None) | Not evaluated (None) | Not evaluated (None) | Not evaluated (None) | Not evaluated (None) |
| I3 (Reference to other metadata) | No (0) | No (0) | No (0) | Yes (1) | Yes (1) | No (0) |
| R1 (Metadata for reuse) | 56 metadata fields (1) | 58 metadata fields (1) | 25 metadata fields (1) | 1001 metadata fields (1) | 17 metadata fields (1) | 7 metadata fields (1) |
| R1.1 (License) | name: other url: <https://www.ebi.ac.uk/arrayexpress/help/data_availability.html> (1) | name: other url: <http://www.ncbi.nlm.nih.gov/geo/info/disclaimer.html> (1) | name: CC0  url : <https://creativecommons.org/publicdomain/zero/1.0/>  name: Copyrights  url : <https://echa.europa.eu/web/guest/legal-notice> (1) | name: Copyrights  url : <https://www.cancer.gov/policies/copyright-reuse> (1) | name: CC  url : <https://creativecommons.org/licenses/by-sa/3.0/>  name: other  url : <https://www.ebi.ac.uk/about> (1) | name: CC  url : <https://creativecommons.org/licenses/by/4.0/>    name: Copyrights  url : <https://ssl.biomax.de/nanocommons/bioxm_portal/bin/view/BioXM/TermsLegal> |
| R1.2 (Provenance) | Authors: Garcia-Esparcia P, Schlüter A, Carmona M, Moreno J, Ansoleaga B, Torrejón-Escribano B, Gustincich S, Pujol A, Ferrer I Email: aschluter@idibell.org Title: Functional genomics reveals dysregulation of cortical olfactory receptors in parkinson disease: novel putative chemoreceptors in the human brain (1) | No (0) | No (0) | No (0) | Authors: Choquenet B, Couteau C, Paparis E, Coiffard LJ.  Title: Journal of natural products (0.5) | No (0) |
| R1.3 (Community standards) | Not evaluated (None) | Not evaluated (None) | Not evaluated (None) | Not evaluated (None) | Not evaluated (None) | Not evaluated (None) |
